# Molecular Crowding: Physiologic Sensing and Control

**DOI:** 10.1146/annurev-physiol-042222-025920

**Published:** 2023-11-06

**Authors:** Arohan R. Subramanya, Cary R. Boyd-Shiwarski

**Affiliations:** 1 Renal-Electrolyte Division, Department of Medicine, University of Pittsburgh School of Medicine, Pittsburgh, Pennsylvania, USA;; 2 Department of Cell Biology, University of Pittsburgh School of Medicine, Pittsburgh, Pennsylvania, USA; 3 Pittsburgh Center for Kidney Research, University of Pittsburgh School of Medicine, Pittsburgh, Pennsylvania, USA; 4 VA Pittsburgh Healthcare System, Pittsburgh, Pennsylvania, USA

**Keywords:** molecular crowding, excluded volume, phase separation, biomolecular condensate, cell volume regulation

## Abstract

The cytoplasm is densely packed with molecules that contribute to its nonideal behavior. Cytosolic crowding influences chemical reaction rates, intracellular water mobility, and macromolecular complex formation. Overcrowding is potentially catastrophic; to counteract this problem, cells have evolved acute and chronic homeostatic mechanisms that optimize cellular crowdedness. Here, we provide a physiology-focused overview of molecular crowding, highlighting contemporary advances in our understanding of its sensing and control. Long hypothesized as a form of crowding-induced microcompartmentation, phase separation allows cells to detect and respond to intracellular crowding through the action of biomolecular condensates, as indicated by recent studies. Growing evidence indicates that crowding is closely tied to cell size and fluid volume, homeostatic responses to physical compression and desiccation, tissue architecture, circadian rhythm, aging, transepithelial transport, and total body electrolyte and water balance. Thus, molecular crowding is a fundamental physiologic parameter that impacts diverse functions extending from molecule to organism.

## INTRODUCTION

Regardless of organism and cell type, all cells harbor a cytosol packed with biologically active molecules. Overcrowding of the cell’s already loaded interior can be life threatening. To defend against this problem, considerable energy is spent recruiting diverse mechanisms that tightly control fluid volume, size, and cytosolic macromolecule concentration. While some of the processes involved are rapid, initiating within seconds of cell volume contraction, others are more chronic, occurring over hours to days to ensure long-term survival.

Because internal fluctuations in cytosolic macromolecule concentration potentially influence a broad array of physiological processes, cells must have mechanisms in place that allow them to sense and respond to an overcrowded environment. Here, we discuss our current understanding of how cells detect macromolecular crowding and how this process impacts cellular response, with a particular focus on cell size and fluid volume regulation. Recent advances in our understanding of cytoplasmic organization have revealed that the phase separation (PS) of proteins into biomolecular condensates constitutes a fundamental crowding sensing mechanism (1). Thus, we also provide a focused overview of the current state of this rapidly expanding field. The macromolecular crowding literature is vast, and a complete discussion of its effects on reaction kinetics is beyond the scope of this review. As this article is primarily focused on the relationship between crowding, biomolecular condensates, and cell physiology, the reader is directed to other excellent reviews that discuss the physical chemistry of crowding in depth (2–4).

## THE CROWDED NATURE OF THE CYTOPLASM

In vitro biochemical studies are usually carried out in solutions that approach ideality, with pure reactants in dilute solutions largely consisting of bulk water. While such efforts are critical for understanding the function of a specific molecule, they are imperfect by design. Physiologically relevant cytosol exhibits the behavior of a nonideal solution, in which its thermodynamic activity is not simply dependent on the concentrations of its individual purified molecular constituents (5). This is due to multiple factors, including the high cytosolic concentration of diverse macromolecules, the decreased availability of free water, and complex spatial organization. Thus, a specific chemical reaction studied with isolated components in the test tube may exhibit differences in biological activity when analyzed in a more physiological context, such as in a native cellular environment.

**Figure 1:**
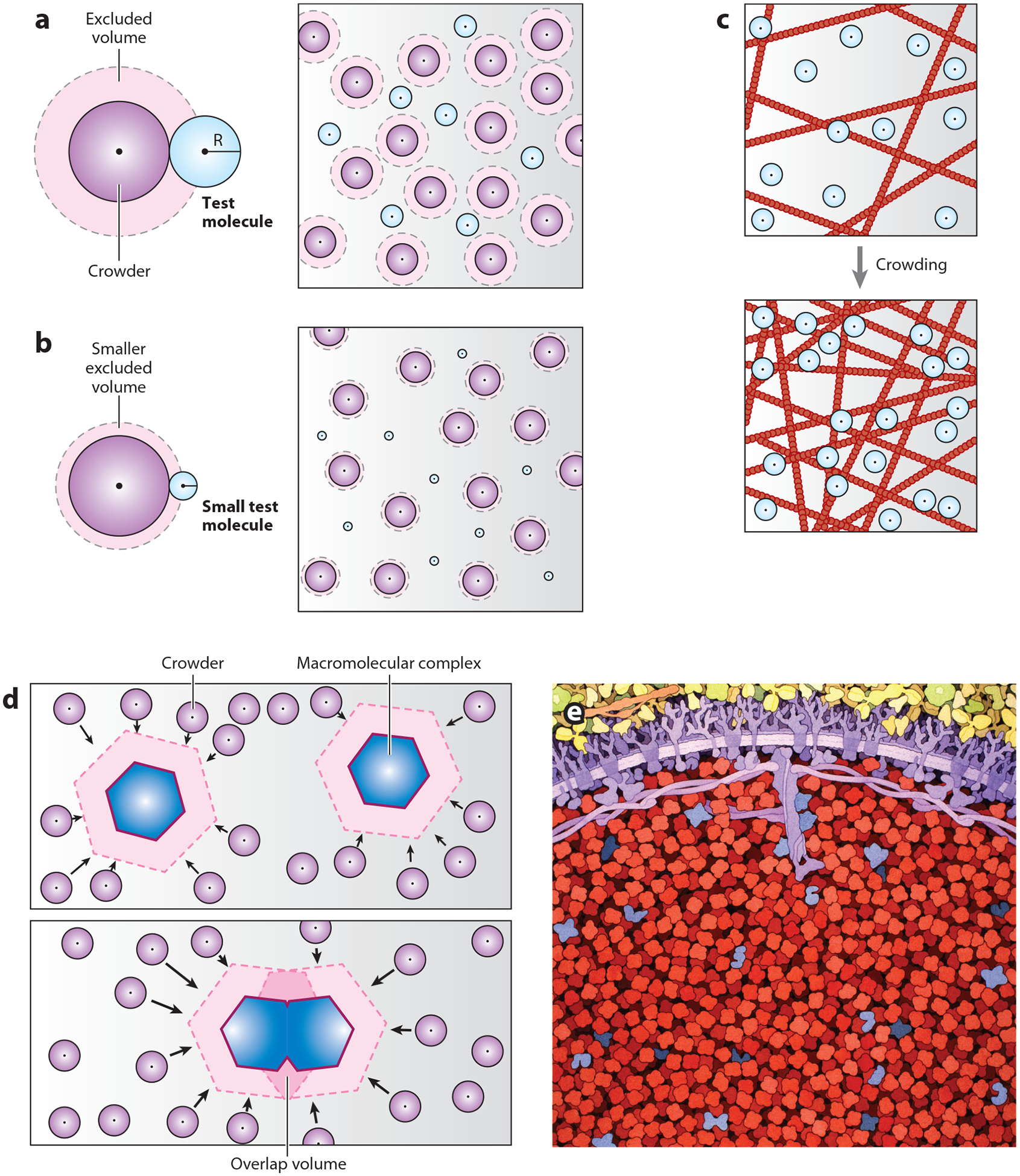
Excluded volume effects, sieving, and depletion-attraction forces. (*a*, *left*) Excluded volume of a cosolvent test molecule in the presence of a macromolecular crowder. The centroid of the test molecule is subject to a volume of exclusion due to steric effects. (*a*, *right*) The test molecule’s volume in which it can move is significantly reduced due to excluded volume effects, resulting in molecular crowding. (*b*) A test molecule of much smaller radius is subject to a smaller volume of exclusion. Thus, the test molecule can move more freely in solution and is less crowded. (*c*) Confinement via cytoskeletal sieving. (*Top*) A population of test molecules (*blue*) in a cytoplasm containing a cytoskeletal matrix (*red*) of large mesh size. The test molecules can easily pass through cytoskeletal pores. (*Bottom*) During molecular crowding, the cytoskeletal pores become significantly smaller, resulting in molecular confinement due to an inability of the test molecules to pass through the mesh (sieving). (*d*) Depletion-attraction forces. (*Top*) Crowders (*purple*) exert forces on large molecular complexes. (*Bottom*) When two interacting complexes bind, their excluded volumes overlap, thereby depleting the total excluded volume in the cell. This increases the hydrostatic pressure of the system, resulting in attraction forces between the two large complexes. (*e*) Simulated view of a structure of a red blood cell, including the large proportion of hemoglobin crowders (*red*). Illustration in panel *e* by David S. Goodsell (CC BY 4.0) (https://doi.org/10.2210/rcsb_pdb/goodsell-gallery-008).

Given the differences between dilute solutions and biological media, investigators sought to understand how nonideal solutions influence protein function. It was from these studies that the concept of molecular crowding emerged. In the 1960s, Laurent & Ogston (6) recognized that concentrated macromolecules exhibit steric excluded volume effects. To understand the concept of excluded volume, consider a homogeneous solution of a single species of well-folded globular (i.e., nearly spherical) monomeric proteins. Two identically sized molecules within this well-mixed solution will bump into one another, but their centers will not overlap, and the minimum distance that can be achieved between two centroids is twice the radius of the spherical protein. Thus, the centroids are subject to a volume of exclusion, which reduces the available space in which they can move. If we add an unrelated test molecule to the solution that is comparable in size, this molecule will similarly be subject to steric interactions that limit its available volume (Figure 1a). Thus, the test molecule will be crowded within specific regions of the solution. These volume-excluded molecules will exhibit altered behavior compared to their dynamics in an ideal solution. For smaller test molecules, the volume of exclusion will be less, and thus the molecule will be able to move more freely in solution (Figure 1b). In the 1980s and 1990s, Allen Minton (7) rigorously tested this concept, developing thermodynamic models that described how background interactions mediated by inert space-occupying macromolecules influence biochemical equilibria and kinetics, and solution nonideality. An important theme that emerged from this work is that biochemical reactions can be either enhanced or suppressed by excluded volume effects (8). Such observations popularized the notion that molecular crowding impacts cellular function and that the inert macromolecules that contribute to this process in vivo effectively function as natural crowding agents.

### Molecular Confinement

An important corollary to the concept of molecular crowding is that of molecular confinement—the microcompartmentation of objects in specific regions in a cell (9). There are likely multiple mechanisms by which molecules may be confined by the effect of molecular crowding. For example, supramolecular matrices such as the cytoskeleton can form lattice-like networks with pore sizes that become compacted during cytoplasmic crowding (Figure 1c). As this process occurs, the movement of molecules may be slowed by steric cytoskeletal interactions through sieving effects (10). Consequently, the kinetics of specific biochemical reactions mediated by those molecules can be altered by several orders of magnitude (11). The slowing effects of molecular sieving scale up with the size of the molecule; although large macromolecules may be nearly arrested, small molecules have the capacity to diffuse more freely throughout the cytoplasm (12). In theory, sieving effects may provide mechanistic connections between crowded physiological signals and cytoskeletal architecture.

Crowders also exert depletion-attraction forces on other cytoplasmic proteins, and this can augment molecular confinement (13). To understand this concept, consider a large object (such as a macromolecular complex) sitting in a solution that contains an overabundance of inert crowding agents (Figure 1d). The crowders will collide with the object and, as two large complexes come closer together, the excluded volumes surrounding them will overlap. Thus, the total volume in which the crowders can move will be increased, which will increase the entropy and osmotic pressure of the surrounding solution. This pressure generates an attractive depletion force that will surround the large molecules, pushing them closer together. In this way, molecular crowding can cause the association and confinement of molecules into clusters, aggregates, or other forms of condensed matter (13, 14). One form of molecular confinement that is likely influenced by depletion-attraction forces and is being increasingly linked to cellular functions is PS, a process by which specific populations of well-mixed cytoplasmic molecules partition into concentrated and dilute phases during stress (15, 16). Molecules within a dense phase can be confined within a space whose biochemical environment differs from baseline conditions in which it is diffusely spread throughout the cytoplasm. Given the emergence of this phenomenon as a thermodynamic response to crowding in vivo, PS and its physiological relevance are discussed in detail below.

Crowding-induced molecular confinement has diverse effects on cellular processes. In many cases, confinement promotes macromolecular interactions between specific binding partners and thus can accelerate reaction kinetics (17). In cases where severe overcrowding is induced, however, confinement can be so excessive that diffusional arrest triggers a slowdown of intracellular signaling (18).

### Crowding and Water Solvent Dynamics

Although the exclusion of molecules from certain regions of the cell is most often viewed from the perspective of steric effects, one can alternatively view crowdedness in terms of how it alters the behavior of the space between macromolecules, i.e., the water that surrounds a crowded object. Consider the zone of exclusion depicted in Figure 1a: While this region is deficient in proteins, it is filled with water molecules. All proteins have a surrounding hydration shell. When water encounters a proteinaceous surface, it can bind to it and organize into a structure. This water, termed interfacial water or water of hydration (19), exhibits a lower degree of entropy, and its activity and mobility are different from that of free-flowing or bulk water (20). Interfacial water is an important component of three-dimensional protein structure and can thus contribute to biomolecular activity (20, 21). In addition, due to its constrained nature, interfacial water is osmotically inactive, whereas bulk water is osmotically active and can therefore shift out of cells during hypertonic stress (22). As the degree of crowding increases in a cell, an increased fraction of water will be bound to protein and, thus, the ratio of interfacial to osmotically active water will increase. This will decrease the amount of free water that can contribute to cytosolic fluidity (23). These effects on fractional interfacial water and on overall viscosity might contribute to cellular dysfunction or physiologic response. Indeed, at least some of the effects of crowding on protein function go beyond standard protein–protein interactions, and instead may be driven by direct effects on intracellular water potential and macromolecular hydration status (134).

### Do Specific Macromolecules in Cells Function as Natural Crowding Agents?

Regardless of whether functional crowders are synthetic or derived from nature, they tend to be large (>50 kDa), globular, soluble, and relatively inert (24). Dextrans, Ficolls, and polyethylene glycols (PEGs) are synthetic crowders that are commonly used to study excluded volume effects in the test tube. Naturally occurring extracellular proteins such as bovine serum albumin and ovalbumin also function as effective crowders in vitro.

Within cells, diverse macromolecules collectively determine crowdedness. However, specific large macromolecules that are more abundant than other cytoplasmic proteins may contribute more significantly to a cell’s ability to exert excluded volume effects. In red blood cells, for example, hemoglobin (a 65-kDa protein) functions as an effective crowder (25). As depicted in Figure 1e, hemoglobin is by far the most abundant macromolecule in red blood cells, comprising approximately 95% of total cellular protein at concentrations exceeding 300 g/L (26). Decades ago, Ross & Minton (27) reported that concentrated solutions of hemoglobin that approximate its abundance in red blood cells have nonideal properties and that these characteristics could be accurately predicted in a mathematical model in which the hemoglobin molecules were depicted as simple nonoverlapping hard spherical particles. In other words, the nonideal properties of hemoglobin-containing solutions can be fully explained by hemoglobin’s ability to function as a crowding agent. More recently, Delarue et al. (28) reported that ribosomes also function as natural crowding agents. Ribosomes are among the most abundant macromolecules in eukaryotic cells. For example, yeast contains approximately 2–4 × 10^5^ ribosomes per cell, which construes about 50% of the total protein copy number (29). Delarue et al. (28) reported that the long-range diffusion of particles in cells was proportional to ribosomal protein concentration, suggesting that ribosomes can function as crowders in vivo. Consistent with this, they also found that purified ribosomes can function as effective crowding agents in the test tube.

### Crowdedness as a Physiological Parameter

Research on the physicochemical effects of macromolecular crowding is extensive and actively growing, and the discussion here is meant to provide an overview for physiologists. However, we would like to highlight some key take home points that are evident from past and present literature. First, it should be obvious at this point that the eukaryotic cytoplasm is crowded, and this gives rise to functional effects that go beyond those which can be reconstituted with select purified components in bulk solvent. Second, crowding leads to the confinement of biological processes, which in and of itself can trigger localized effects in the cytoplasm. Third, crowding can either amplify or dampen chemical reactions through diverse effects that modulate protein–protein and protein–solvent (water) interactions. And fourth, due to their abundance, size, and other features, the contributions of certain proteins to overall cellular crowdedness exceed that of others. Thus, the expression of specific genes dictates crowding status. Because crowding influences diverse cellular processes and is under cellular control, these observations collectively indicate that molecular crowding is a fundamental cellular property that undergoes physiological regulation. Conceivably, the physiological scenarios in which intracellular crowding might be influenced are vast, ranging from acute tonic or isosmotic shifts in fluid volume, to the overpacking of cells within a confined space due to proliferation or changes in tissue architecture (30) and to compression caused by mechanical forces.

## HOW DO CELLS SENSE MOLECULAR CROWDING?

### Confinement as a Cell-Sensing Mechanism

Because cells can regulate their crowdedness, they must possess mechanisms that permit the detection of crowding, and this needs to be coupled to their capacity to mediate crowd control in response to stress, both acute and chronic. The importance of molecular confinement (also referred to as microcompartmentation) was recognized early on as a potential way in which cells could react to overcrowding. The work of Minton and others suggested that hindered molecular diffusion caused by crowding promotes functional protein–protein interactions that assemble complexes and alter biochemical reaction rates (31, 32). Early experiments also revealed that the molecular density within a crowded cell is not homogeneous (12). Rather, proteins appeared to be localized to certain regions of the cytosol, suggesting that such localization might be the manifestation of specific functions. For example, one hypothesis was that protein interactions with cytoplasmic matrix proteins might be enhanced by crowding, leading to regional effects on cytoskeletal function (33). Alternatively, the binding of specific enzymes to cellular surfaces might organize a metabolon that coordinates a complex process, such as metabolic channeling (34). Localized microcompartmentation of specific molecules near the membrane was also proposed to couple crowding to membrane protein function (9). By and large, however, despite decades of interest in the physicochemical effects of crowding, the mechanisms by which cells sense a crowded environment remained poorly understood.

### Phase Separation Drives Molecular Confinement

**Figure 2:**
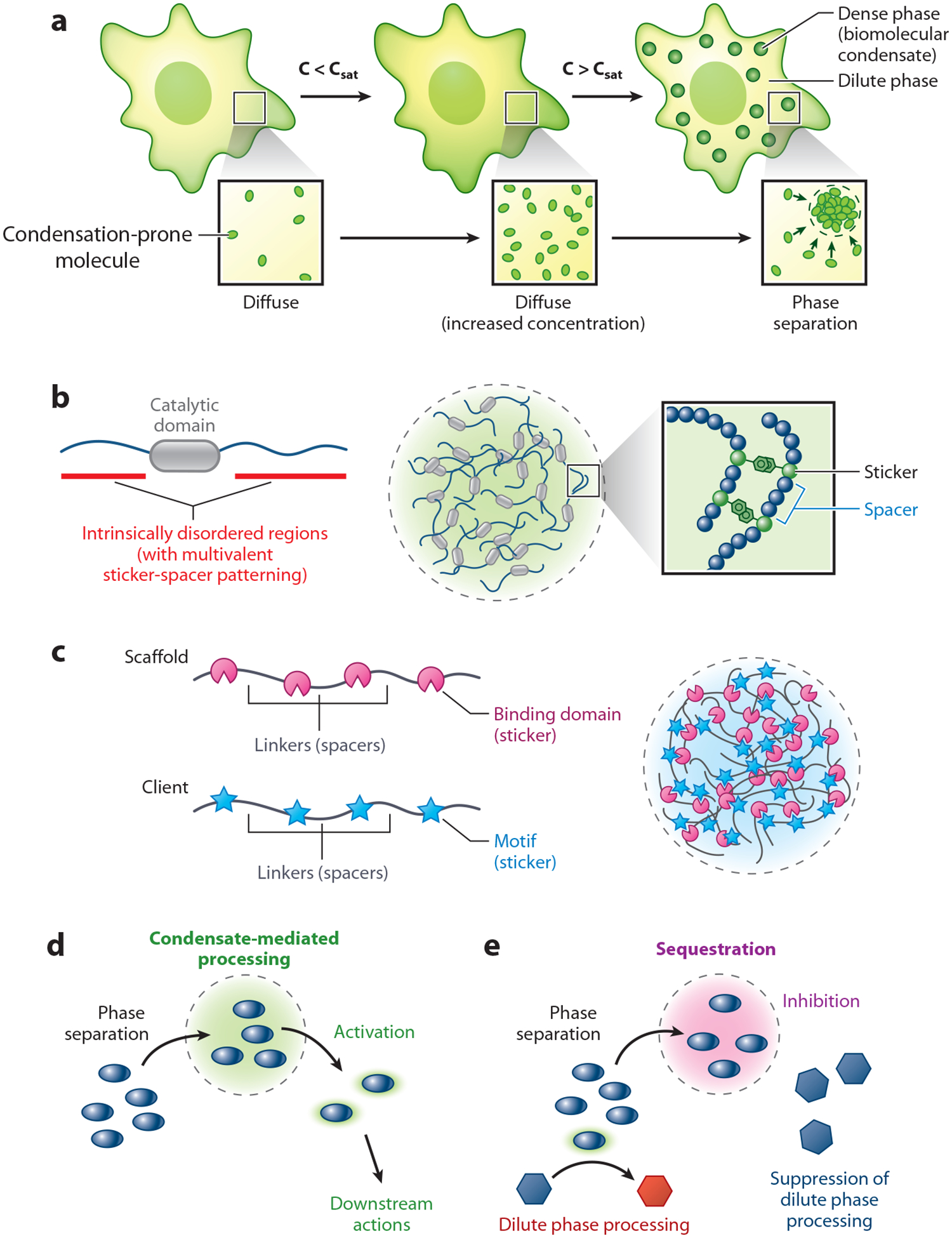
Phase separation (PS) and biomolecular condensates. (*a*) Depiction of a cell containing a population of molecules prone to PS. If the concentration of molecules is below c_sat_ (the saturation concentration that is required for PS), they will be diffusely spread throughout their cellular compartment. If the concentration of molecules increases to above c_sat_, they will partition into two phases. The dense phase manifests as punctate assemblies termed biomolecular condensates that lack a surrounding membrane. As these structures are concentrated in the phase-separated molecule, the surrounding dilute phase will be relatively depleted of those molecules. (*b*,*c*) Sticker-spacer model for multivalent proteins. (*b*) Shown is a phase-separating molecule containing a catalytic domain flanked by two intrinsically disordered regions (IDRs). The IDRs mediate sticker-sticker interactions between aromatic residues through weak Pi-Pi stacking effects. The stickers are separated by spacers of a limited number of residues. Sticker number (valence) and positioning relative to spacers (patterning) are critical for optimal phase behavior. (*c*) A scaffold molecule for phase transitions containing a multivalent array of folded binding domains separated by linker regions interacts with a multivalent protein containing binding motifs separated by linkers. Here, the folded domains and motifs function as stickers, while the linkers are spacers. Heterotypic sticker-sticker interactions result in PS. (*d*,*e*) Signal processing by biomolecular condensates. (*d*) Biomolecules can undergo processing within condensates, resulting in their activation. Because molecules exchange between dense and dilute phases following processing, they can leave the condensates to influence downstream processes (1). (*e*) Conversely, some signals can be sequestered within condensates, preventing them from regulating processes occurring outside of the dense phase (65, 100).

In recent years, there has been a paradigm shift in our understanding of how cellular space is organized. This has been driven by a large and rapidly expanding body of evidence demonstrating that many molecules within the cell undergo a microcompartmentation process called PS (15). PS is a physiochemical process in which molecules are forced into two phases; one of these phases is enriched in specific molecules, whereas the other is depleted of those same molecules. Biomolecular PS is a concentration-dependent process (35). Normally, molecules are beneath the saturation concentration that is required for PS, a threshold referred to as c_sat_. When the concentration of a biomolecule is less than c_sat_, it will be diffusely spread throughout its cytoplasmic compartment. When the concentration increases above c_sat_, however, it will separate into dense and dilute phases (Figure 2a). The dense phase often contains greater than hundreds of distinct molecules, manifesting as punctate mesoscale structures whose volumes are larger than nanoscale clusters but smaller than large cellular objects spanning several micrometers. Thus, the dense phase consists of puncta that are often (but not always) detectable with conventional microscopes. These condensed assemblies lack a surrounding membrane and can have liquid-like characteristics, as they contain dynamically mobile material, merge into spherical droplets, and wet against surfaces. For this reason, the terms phase separation and liquid–liquid phase separation (LLPS) are often used interchangeably. The reader should be aware, however, that PS can result in the formation of a dense phase containing networked and/or dynamically arrested proteins that exhibit more solid or gel-like properties (15). Moreover, the composition of a liquid-like condensed phase is more complex than a simple liquid (36), as it can exhibit viscoelastic properties that age, or change over time (37). Specific molecules drive PS, forming assemblies that have distinct compositions; this suggests they mediate specific functions. Consistent with this, molecules can enter a dense phase, undergo processing, and subsequently reenter the dilute phase (15). Indeed, PS has been implicated in the formation of various membraneless organelles in cytoplasm and nucleoplasm, including nucleoli, Cajal bodies, centrosomes, stress granules, and P bodies. Such structures have been collectively termed biomolecular condensates to emphasize the concept that PS concentrates specific biological phenomena within a microcompartment (38).

The concentration of a specific biomolecule is not the only parameter that dictates PS; the stress that a biomolecule is subjected to is also a factor. Multiple biological stressors can drive protein PS in cells, including changes in pH (39), cytosolic DNA (40), and temperature (41). This suggests that proteins and other molecules phase separate as part of an environmental stress-sensing and response mechanism (42, 43). From a functional perspective, the identification of PS as a biological stress-sensing mechanism has broad implications for understanding how signals are coordinated in response to physiological stimuli.

### Molecular Crowding and Phase Separation

Crowding also drives stress-induced biomolecular condensate formation (1, 44, 45). The concept that macromolecular crowding might serve as a stimulus for in vivo PS was first proposed decades ago. In 1971, for example, Lerman (46) observed that in vitro, DNA formed dense particles in the presence of synthetic crowders and speculated that this process was due to a structural phase transition driven by conditions that mimicked the crowded environment of cells. In a 1995 essay, Walter & Brooks (47) expanded upon this concept, hypothesizing that intracellular crowding is likely sufficient to cause diverse biomolecules to undergo PS. They suggested that excluded volume effects induced by molecular crowding in vivo might sufficiently increase the effective concentrations of molecules, forcing them to partition into dense and dilute phases, even at native expression levels. This crowding-induced localization within dense phases would be expected to promote cytosolic inhomogeneity, restrict diffusion, and concentrate molecules that mediate specific biochemical reactions, thus fulfilling the proposed effects of confinement on biological processes (9). Thus, PS could be the biophysical manifestation of a functional response to crowding.

The theory of crowding-induced PS as a form of microcompartmentation remained underappreciated until contemporary interest in biomolecular condensates increased dramatically in 2009, following the discovery that a membraneless organelle in *Caenorhabditis elegans* exhibited liquid droplet–like properties (48). Numerous subsequent studies employed in vitro approaches to evaluate how specific biomolecules exhibit liquid phase behavior. While some of those studies identified proteins that phase separate in ideal solutions, most do not readily phase separate under such conditions, unless their concentrations are increased far beyond the physiologic range. Thus, as studies on PS progressed, investigators sought to evaluate in vitro PS in environments that mimic the cytoplasm, which led to the use of crowding agents. In most cases, adding synthetic crowders to a reaction mixture increases the ability of a specific biomolecule to phase separate (44); in vitro studies with natural ribosome crowders have supported this finding (28). As a result, contemporary in vitro studies of biomolecular condensates routinely employ crowding agents to model phase behavior.

### Why Do Molecules Phase Separate?

Numerous different types of biomolecular condensates exist within cells, and their molecular compositions are diverse (38). In each case, condensates will contain specific molecules that drive the formation of a condensed phase. Common themes have emerged from the study of proteins that function as PS drivers. One consistent feature is multivalency: the presence of multiple (or repeating) specific modules that facilitate protein–protein or protein–nucleic acid interactions (49). Depending on the protein and presumably the type of condensate it assembles, these multivalent interactant modules can come in a variety of forms, such as a folded domain [e.g., an SH3 domain (50)], a specific linear amino acid motif [such as the phenylalanine-glycine repeats in nucleoporins (51, 52)], or recurrent patterns of charged or aromatic amino acids (53, 54). Although some of these interactions (such as folded domain interactions) may be due to specific binding events, others such as those conferred by multivalent patterned residues are less sequence specific and can be phenocopied by replacing the original configuration of amino acids with different residues that share similar biochemical properties. In general, however, the multivalent interactions that drive LLPS are thought to be weak and/or transient associations (15). Thus, more stringent biochemical methods that are classically used to identify protein–protein interactions, such as coimmunoprecipitation assays, may not be able to detect such associations.

Proteins that phase separate also contain areas that tend to be enriched in specific amino acids (55). These low-complexity regions (LCRs) are often intrinsically disordered and thus do not fold into a single specific conformation. Intrinsically disordered LCRs often contain prion-like domains—aggregation-prone stretches of amino acids initially identified in prion proteins that contribute to pathogenicity (56). However, these prion-like regions are present throughout the healthy proteome, and it is now well appreciated that they play a key role in determining the form and function of biomolecular condensates required for normal physiologic function (39, 57).

Insights into how intrinsic disorder and valence contribute to protein PS have been incorporated into a generalized stickers-and-spacers model (58). This model provides a versatile and testable framework for exploring the mechanistic basis of PS, as it rationalizes the phase behavior of intrinsically disordered regions (IDRs) that engage in homotypic interactions, while it also explains how proteins can partition into heterotypic multicomponent phases with binding partners. In both scenarios, the multivalent modules function as stickers that mediate associative interactions, whereas the spacers reside in between the stickers and preferentially interact with solvent. In the case of an isolated phase-separating IDR, the stickers are individual amino acids in the disordered region that are attracted to amino acids on a partner protein through specific low-affinity noncovalent interactions (e.g., cation-pi, pi-pi, charge, or dipole interactions) (Figure 2b). A prototypical example of this would be the tyrosine and phenylalanine stickers in the prion-like LCRs of the precursor messenger RNA (pre-mRNA) processing protein heterogeneous nuclear ribonucleoprotein A1 (hnRNPA1) (53). These residues feature aromatic ring sidechains that engage in noncovalent pi-pi interactions with aromatic residues on an hnRNPA1 binding partner; these attractive forces drive PS (59). The low-complexity spacers in this example largely consist of glycine and serine amino acids that are interspaced between the aromatic stickers in tracts of <10 residues. This interval spacing pattern seems to be critical for the phase behavior of the domain, as altering the arrangement of stickers and spacers changes the material properties of the hnRNPA1 condensates. This suggests that while spacers discourage the driving force for PS due to preferential solvent interactions while a molecule is diffusely spread throughout the cytosol, once inside a condensate they may be critical for controlling condensate fluidity (rheology) (58). As these interactions are occurring among a homogeneous in vitro population of hnRNPA1 molecules in solution, this constitutes a form of homotypic PS. The stickers-and-spacers model can also describe heterotypic PS with two different proteins with folded domains that bind linear motifs (Figure 2c). A proof-of-principle example for such an interaction is a multivalent SH3 domain–containing protein binding to a partner harboring an array of proline-rich motifs (50). In this example, the SH3 domains and proline-rich sequences function as stickers that are spread across their respective polypeptide sequences by linkers, which function as spacers. When incubated in solution, both of these model proteins phase separate into the same condensate, and the valence (i.e., the total number of stickers on each protein) modulates the efficiency of phase behavior. These in vitro insights were critical for the development of the sticker-and-spacer model and provide a foundation for future studies that will test the mechanisms of biomolecular PS in a more physiologic context.

### Phase Separation as an Overcrowding Stress-Sensing Mechanism

Taking the sticker-and-spacer model into account, one can envision how proteins might phase separate in response to cellular overcrowding. The excluded volume effects of crowding (Figure 1a) would essentially increase the effective concentration of molecules that engage in homo- or heterotypic sticker-sticker interactions. At the same time, the loss of bulk cellular water would reduce spacer-solvent interactions that discourage PS. The result is that the effective concentration of PS-prone proteins will rise above c_sat_, triggering the formation of a condensed phase. Once a population of condensates is established amid a crowded environment, it is conceivable that depletion-attraction forces generated by overlapping excluded volumes (Figure 1d) might cause condensates of identical compositions to come into contact, fuse, and resolve into spherical viscoelastic droplets. The interplay between protein phase behavior and excluded volume effects suggests that PS functions as an overcrowding sensing mechanism in cells. Indeed, it provides an elegant solution to the molecular confinement and microcompartmentation hypothesis that was popularized by scientists decades ago (12).

### Diverse Molecules Phase Separate in Overcrowded Conditions

Consistent with the notion that PS functions as a generalized overcrowding stress response, the list of proteins that exhibit crowding-associated phase behavior in vivo is expanding. Most studies that have identified in vivo biomolecular PS under crowded conditions have done so by acutely subjecting cells to a hypertonic extracellular environment. As cells exposed to hypertonicity rapidly shrink due to exosmosis, their internal cytoplasmic milieu becomes crowded (60). Numerous molecules have been shown to form punctate foci under these conditions. Jalihal et al. (61) used a combination of high-throughput immunofluorescence and transient green fluorescent protein–tagged protein overexpression to test the effect of hypertonicity on the localization of greater than 100 proteins and reported that many of these proteins formed puncta (presumably condensates) following stress. Proteins that changed localization tended to favor multivalent interactions, as many of them contained either LCRs or multimerization domains. Proteins that have been more extensively interrogated in cells and reported to undergo PS in response to hypertonicity include P body and stress granule constituents (61), transcription factors (62), cochaperones (63), proteasome subunits (64), DNA repair components (65), kinases (1, 66), and numerous other examples. Thus, biomolecular condensates participate in diverse crowding-dependent cellular processes.

### Crowding-Dependent Signal Processing Within Biomolecular Condensates

As discussed above, crowding-mediated microcompartmentation can alter the rate of biochemical reactions. Consistent with this, biomolecular condensates can exert similar effects. Condensates can function as reaction hubs that accelerate cellular reactions (Figure 2d) or as bodies that sequester molecules, preventing them from becoming activated (15) (Figure 2e). Current evidence indicates that as molecules within a signaling pathway become confined within biomolecular condensates, their activities can change severalfold due to effects on molecular organization/scaffolding, substrate *K*_m_, and mass action (67). For example, in the case of a synthetic kinase signaling cascade that forms phase-separated designer condensates in cells, application of a hypertonic 1 M sorbitol stress caused cells to compress, which rapidly decreased cytoplasmic fluidity and increased the estimated ribosome crowder concentration from 23 to 32 μM (45). This was associated with hyperphosphorylation of the synthetic pathway within the condensed phase. When various perturbations were applied to the system to deplete ribosomes, the pathway was less active. Thus, kinase activation was dependent on the degree of macromolecular crowding.

Phosphorylation-dependent processing of molecules within condensates can be associated with altered phase behavior. Serine residues are frequently present in low-complexity IDRs (55). If a PS-driving IDR preferentially condenses when its serines are in the unphosphorylated state, phosphorylation within condensates would alter the domain’s charge, generating repulsive forces that drive it out of condensates and back into the dilute phase. This has been reported for certain IDR-containing PS drivers that undergo phosphorylation in condensates, such as the LCR of the RNA-binding protein fused in sarcoma (68). Thus, biomolecular condensates are dynamic structures in which material enters and leaves in concert with post-translational processing, making them regulators of cellular signaling in response to stressors such as overcrowding.

### Condensates Are Not Without Their Controversies

The field of condensate biology is rapidly expanding to the point where membraneless assemblies have been implicated in biological processes that span the full spectrum of evolution. Despite great excitement, however, condensates have also been the subject of intense debate and skepticism, as has been well summarized in recent perspective and news articles (69–73). Key issues identified include the reductionist nature of in vitro studies that fail to capture the richness and complexity of multicomponent condensates in living systems (70, 71), poor quality in vivo characterization of stress-induced foci/puncta as nonmembrane-bound compartments (70), suboptimal rigor in direct tests of PS dependency on a biological process (71, 73), the lack of attention to physiologic range when studying PS-associated phenomena (70), and the lack of clear connections to a bona fide functional response (70).

Many efforts to connect biomolecular condensates to functional outputs are focused on transcriptional responses or biochemical activity. Although such experiments might support a condensate-dependent role in dynamic signal transduction, efforts to follow through all the way to a bona fide physiological response are often lacking. Such functional outputs may occur on timescales of hours to days, making them difficult to align with rapid phase behavior that occurs over seconds to minutes. In the case of some membraneless organelles such as stress granules or P bodies, the biological function of the condensate is still poorly understood, making it challenging to study a clear downstream effect (74). Moreover, while in vitro experiments may suggest a role for condensates in signal amplification under extreme conditions, the effect size may be small when tested within the physiological range of a cell or organism. Thus, though stress may drive PS of a specific biomolecule, the condensate that it forms may not be physiologically impactful.

In summary, to understand the biological significance of PS and biomolecular condensates, clear connections to real physiologic outputs are needed. In the next section, we discuss how one such functional output—cell volume regulation—was recently recognized as a PS-dependent process that occurs through the sensing and control of molecular crowding.

## REGULATION OF MACROMOLECULAR CROWDING

### Cell Volume Regulation and Crowding

Cells cultured in an isotonic medium exhibit a stable fluid volume. Due to significant differences in intracellular and extracellular content, however, cells are constantly working to maintain this equilibrium. The crowded intracellular milieu, largely consisting of charged proteins, creates an inequity that needs to be counterbalanced. These fixed macromolecules carry a net anionic charge, which creates attractive forces for positively charged cations, mainly sodium and potassium, creating a Donnan effect (75). There is another problem that cells need to address, however: the colloid osmotic pressure that is generated by the fixed population of intracellular proteins. This creates an osmotic driving force for water to enter the cell, and if cells did not have a way to address this, they would eventually swell and burst. To counter this problem, cells evolved pumps that move osmotically active cations back across the membrane (76, 77). In most cells, this function is mediated by the Na^+^/K^+^ ATPase, which pumps out Na^+^ in exchange for K^+^ influx at a ratio of 3:2, creating a second Donnan potential. Thus, isotonic cell fluid volume is dictated by a double Donnan effect, which is dependent on a combination of membrane ion permeabilities, cytoplasmic protein concentration (crowding), and counteracting pumps (78, 79). This indicates that crowding is a physiologic parameter that cells control.

During acute shrinkage, overcrowding can potentially perturb every biochemical reaction in a cell (80). To avoid such a catastrophe, cells harbor fail-safe mechanisms that initiate fluid volume recovery within seconds of hypertonic stress (81). These evolutionarily conserved processes involve the coordinated effects of ion transporters, which drive the net import of cations, chloride, and water, a process termed regulatory volume increase (RVI). Though essential for cellular health, RVI only functions as a temporary fix, giving cells an opportunity to synthesize osmolytes or initiate processes that adjust endogenous crowder concentration and/or size to ensure long-term viability. In 1991, Cossins (82) summarized the work of several investigators that defined a phosphorylation-dependent RVI ion transport system in red blood cells. Hypertonic stress appeared to activate an electroneutral Na^+^/K^+^/2Cl^−^ transporter that mediates influx, while simultaneously shutting off electroneutral K^+^/Cl^−^ efflux via a different transporter. This net influx of ions was associated with the RVI response. The effect was dependent on phosphorylation, suggesting the involvement of a kinase signaling pathway in the volume recovery mechanism.

A major question of interest at the time was how red cells are capable of sensing acute volume loss to trigger the RVI signal (83). To answer this question, Parker & Colclasure (84) designed a series of experiments in ghosts, red cells that were lysed and subjected to resealing conditions that manipulated their size and crowder composition. Their experiments revealed that irrespective of cell size or type of crowder that was used, the volume regulatory responses to hypertonic shrinkage or hypotonic swelling were dependent on the intracellular concentration of crowding agent. Based on these results, they concluded that osmotically stressed red cells indirectly sense changes in cell volume by monitoring the degree of cytoplasmic crowding. Working with Allen Minton, they developed a mathematical model of transport that recapitulated their experimental results (25). These findings provided physiological support for Zimmerman & Harrison’s speculation in 1987 that “changes in reaction rates due to changes in crowding provide, in principle, a simple mechanism by which the cell could sense changes in its own volume” (85, p. 1875).

### The WNK-SPAK/OSR1 Pathway and Crowding Sensing

We now know that the RVI transport mechanism summarized by Cossins (82) is mediated by specific members of the SLC12 family of electroneutral cation chloride cotransporters (86). Hypertonic stress activates the Na-K-2Cl cotransporter NKCC1 (*SLC12A2*) and inhibits K-Cl efflux via a subfamily of four KCC cotransporters (KCC1–4; *SLC12A4*–*7*). These NKCC and KCC cotransporters are phosphorylated by a common serine-threonine kinase cascade that requires with-no-lysine (WNK) kinases (86). Hypertonic stress activates the WNKs, which activate two downstream kinases, Ste20-like proline-alanine-rich kinase (SPAK; *STK39*) and oxidative stress–responsive kinase 1 (OSR1; *OXSR1*) (87). Upon activation, SPAK and OSR1 directly phosphorylate the NKCC and KCC cotransporters, resulting in the net influx of ions and water, which drives volume recovery (88, 89).

**Figure 3:**
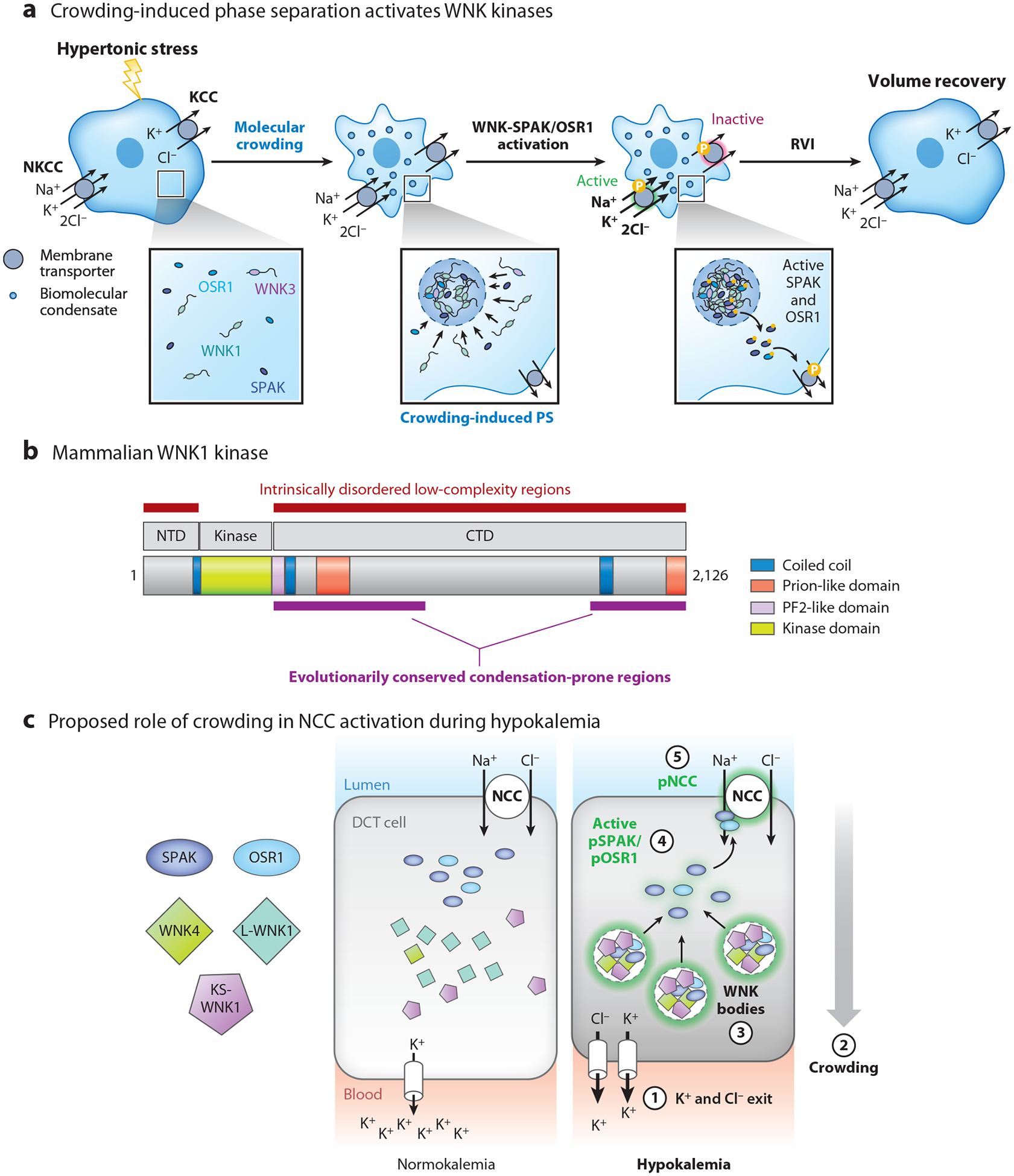
WNK kinases are crowding sensors that regulate physiology via phase transitions. (*a*) During cell shrinkage induced by hypertonicity, WNK kinases undergo PS, activating the downstream kinases SPAK and OSR1, which phosphorylate NKCC and KCC ion cotransporters. This results in the activation of net ion influx and volume recovery (1). (*b*) PS of WNK kinases occurs via the CTD, which functions as a crowding-sensing domain. The CTD is an evolutionarily conserved large IDR that contains two coiled coils and prion-like regions. The entire CTD is required for WNK kinases to sense physiologic changes in molecular crowding. (*c*) Proposed role of crowding in WNK body formation in DCT epithelia during hypokalemia. Schematic depicts DCT cells at different states of systemic K^+^ balance. During normokalemia, the WNK-SPAK/OSR1 pathway is spread diffusely throughout the cytosol. During hypokalemia, ① basolateral K^+^ and Cl^−^ exit triggers localized volume contraction, ② molecular crowding on the basal side of DCT cells, which generates a polarized crowding gradient, ③ crowding-induced WNK body condensate formation, ④ pathway activation, and ⑤ NCC activation via phosphorylation (p). Abbreviations: CTD, C-terminal domain; DCT, distal convoluted tubule; IDR, intrinsically disordered region; KCC, K^+^−2Cl cotransporter; KS, kidney-specific; NCC, Na-Cl cotransporter; NKCC, Na^+^−K^+^−2Cl cotransporter; NTD, N-terminal domain; OSR1, oxidative stress–responsive kinase 1; PS, phase separation; RVI, regulatory volume increase; SPAK, Ste20-like proline-alanine-rich kinase; WNK, with-no-lysine kinase. Panels *a*, *b* adapted with permission from Reference 1; copyright 2022 Elsevier. Panel *c* adapted from Reference 110.

Though it was well appreciated that the WNK-SPAK/OSR1 pathway triggers NKCC/KCC phosphorylation (90) and RVI (91), the mechanism of pathway activation during hypertonic stress was poorly understood until recently. Current evidence indicates that hypertonicity triggers crowding-induced PS of the WNK kinases, forming membraneless biomolecular condensates that activate SPAK and OSR1 (1) (Figure 3a). Within this system, the WNKs function as PS drivers, as they contain a massive (>100 kDa) intrinsically disordered C-terminal domain (CTD) that is required for the phase behavior. The C-terminal IDR of mammalian WNK1 contains two prion-like regions at its proximal and distal ends, positioned next to coiled-coil domains (Figure 3b). These regions drive PS, with the coiled coils acting to facilitate kinase retention within the condensed phase to augment activation. Notably, the CTD drives PS within physiological constraints, forming visible condensates at native level expression and with hypertonic stresses as low as 25 mOsm above isotonicity (1). SPAK and OSR1 function as clients that enter the condensed phase with the WNKs, leading to their activation within seconds. Approximately 5 min after hypertonic stress, the condensates start to dissolve. Though the mechanism by which this occurs is currently unresolved, this process allows active SPAK and OSR1 to engage with NKCC1 and the KCCs at the plasma membrane to directly phosphorylate the cotransporter, activate net ion influx, and mediate RVI (Figure 3a). In the absence of its C-terminal IDR, the WNK1 kinase domain fails to visibly phase separate, and this impairs hypertonic SPAK/OSR1 activation, leading to reduced NKCC-/KCC-mediated RVI. Remarkably, however, the phase behavior and volume regulatory effects of the WNK1 kinase domain can be nearly fully rescued if the PS-driving IDR of an unrelated protein is appended to it. This indicates that PS of the WNK1 kinase domain is required for efficient RVI (1, 92). Finally, to specifically test the effects of crowding on WNK1 phase behavior, a series of experiments were performed in which the crowding agent Ficoll was microinjected into cells. Despite the increase in cell size caused by injected material, WNK1 underwent robust PS, confirming its role as a physiologic crowding sensor (1). Collectively, these findings support the role of PS in the homeostatic regulation of cell volume and molecular crowding. WNK biomolecular condensates and/or cell volume and crowding-dependent regulatory effects on SLC12 cotransporters have been suggested to play a role in renal tubular transport (93), circadian rhythm (94), brain osmosensing and vasopressin secretion (95), stroke (96), and choroid plexus function (97). This suggests that the crowding-dependent regulation of the WNK signaling pathway has implications for physiological regulation of ion transport in diverse contexts.

Interestingly, the crowding-sensing function of WNK kinases appears to be distinct from their other sensing functions. WNK kinases are also known sensors of intracellular chloride concentration (98). This is mediated by binding sites for chloride located within the kinase domain (99). Recent data also suggest that the WNK kinase domain is regulated by intracellular potassium (100). In both cases these ions inhibit kinase activity. During hypertonic stress, exosmosis increases intracellular ionic strength, which should augment the inhibitory effects of chloride and potassium on the pathway. Because the WNK-SPAK/OSR1 pathway is activated during hypertonic stress, PS of the WNK signaling pathway serves to bypass the inhibitory effects of ionic strength. This may be due to simple mass action effects within the condensed phase or other factors such as an alteration in intracondensate chloride and potassium concentrations. Data also suggest that osmotic pressure dehydrates the WNK kinase domain; these effects on interfacial water content might lead to conformational changes that activate the pathway (101). Whether such effects are occurring within crowding-induced WNK condensates is unknown but seems plausible, especially given prior reports that condensed phases can have altered solvent environments (102).

### Crowding-Dependent WNK Signaling in Polarized Epithelia

WNK kinases are ancient cell volume regulators; their disordered crowding-sensing domains are evolutionarily conserved to protists (1). With the emergence of polarized transporting epithelia in early metazoans, preexisting crowding-dependent cell volume regulatory mechanisms were likely co-opted to drive transcellular transport. As ions leave one side of a polarized cell, water will follow, and this will result in localized macromolecular crowding near the site of ion exit. This in turn can propagate signals that drive ion entry across the opposite membrane to maintain cell volume while facilitating vectorial ion flux to regulate whole animal physiology. WNK kinases are a prime example of how a cell volume regulatory system can be leveraged for such a purpose. For example, the *Drosophila* Malpighian tubule harbors a WNK-SPAK pathway that activates basolateral-to-apical potassium secretion via an NKCC1 ortholog (103). Like the mammalian WNKs, the fly WNK kinase has a large C-terminal IDR that drives robust PS (1). Thus, crowding-induced phase behavior likely facilitates WNK-dependent transport processes in epithelia.

In mammals, one example epithelium that likely leverages crowding-dependent WNK signaling and phase behavior to activate transcellular ion transport is the kidney’s distal convoluted tubule (DCT) (104). WNK kinases are highly active in this nephron segment, as they drive the phosphorylation-dependent activation of NCC (*SLC12A3*), a sodium chloride cotransporter closely related to NKCC1. WNK-dependent NCC phosphorylation is strongly activated during hypokalemia (105). As blood potassium levels fall, the DCT-expressed WNK-SPAK/OSR1 pathway condenses into specialized biomolecular condensates termed WNK bodies (93) (Figure 3c). These membraneless punctate foci contain WNK4, SPAK, and OSR1, thus serving as signaling hubs that regulate pathway activity. Though WNK bodies contain kinase-active WNK1 (sometimes referred to as long WNK1 or L-WNK1) (106), their assembly is instead driven by KS-WNK1, a truncated kidney-specific WNK1 isoform that is highly expressed in DCT (107). KS-WNK1 lacks catalytic activity due to the absence of a functional N-terminal WNK1 kinase domain, but it retains the entire disordered C-terminal crowding-sensing domain that drives phase behavior. Thus, KS-WNK1 scaffolds WNK-dependent phase transitions that activate the DCT-specific WNK-SPAK/OSR1 pathway. During hypokalemia, potassium and chloride exit cells, causing them to shrink (108). When potassium levels are low in the blood, K^+^ and Cl^−^ also leak across the DCT basolateral membrane into the peritubular fluid (109). This likely induces localized molecular crowding near the basolateral membrane, which could drive a phase transition that drives WNK body assembly via a polarized crowding gradient (Figure 3c). Consistent with this, hypokalemic WNK bodies invariably form along the basal aspect of DCT cells, positioned between the nucleus and basolateral membrane (93, 110), and are absent in mice lacking Kir4.1, the basolateral K^+^ channel that facilitates hypokalemic K^+^ efflux in the DCT (111). Thus, current evidence suggests that WNK-dependent phase transitions are not only relevant for general cell volume regulation, but they also activate transcellular ion flux, possibly through crowding-dependent mechanisms.

## ASK3

Crowding-induced PS has been implicated in the regulation of ASK3 (*MAP3K15*), a serine-threonine kinase that also participates in cell volume regulation (112). Unlike the WNKs, which become hyperphosphorylated during cell shrinkage (87), ASK3 phosphorylation is decreased under the same conditions. During hypertonic stress, ASK3 undergoes PS, forming inactivating biomolecular condensates (66). Though the role of condensate-mediated ASK3 suppression on cell volume regulation was not explored in this study, prior work indicates that the ASK3 inhibition is required for optimal RVI following exposure to hypertonic stress (112) and that it functions as an upstream inhibitor of the WNK-SPAK/OSR1 pathway (113). This suggests that the sequestration of ASK3 into a condensed phase prevents it from interfering with WNK-mediated activation of NKCC1 during crowding. Consistent with this, a recent study reported that hypertonic stress-induced ASK3 and WNK1 signaling puncta do not colocalize (114). Thus, it appears that crowding can induce the formation of functionally distinct biomolecular condensates that mediate different aspects of the same stress response.

### Cellular Control of Crowding Agent Abundance

The mechanisms detailed above provide examples of how intracellular crowding can be regulated by changing the intracellular fluid volume that makes macromolecules more dilute or concentrated. However, crowd control can also be accomplished by adjusting the total population of molecules that mediate crowdedness. An example of this was reported in the study by Delarue et al. (28), which identified ribosomes as natural crowding agents. They also found that cellular ribosome concentration could be regulated by the mammalian target of rapamycin complex 1 (mTORC1), a eukaryotic nutrient sensor. Inhibition of mTORC1 increased cytoplasmic fluidity due to decreased ribosome abundance. Rather than a generic effect on cellular function, such as changes in translation or protein degradation, the effect appeared to be specifically tied to a transcription factor that drives ribosome biogenesis. This is consistent with the notion that cells specifically control the abundance of natural crowding agents to tune their responsiveness to crowding. The timescale for such regulation is significantly longer than the RVI mechanism described above, suggesting that cells can regulate crowding in both the short and long term. mTORC1 has been implicated in numerous processes important for cellular health, including the cell cycle, autophagy, cell and animal size, and metabolism. Recently, the complex has also been proposed to provide connections between long-term NKCC1-mediated control of cell fluid volume and total cellular mass (115). These findings suggest intriguing connections between the regulation of molecular crowding, ion transport, and diverse cellular metabolism.

## BEYOND ACUTE OSMOTIC SHOCK: OTHER CROWDING-DEPENDENT PHYSIOLOGIC PROCESSES

### Desiccation

Acute hypertonic shock is a straightforward example of how sudden changes in the extracellular milieu can rapidly augment crowding and its related downstream stress responses. However, hypertonicity-induced crowding is also tied to other more chronic and extreme physiologic stresses such as desiccation—the process by which water is extracted from a system to the point of dryness. Desiccation tolerance is a trait present in certain plants, microbes, and animals [such as tardigrades (116)]. During the process of desiccation, these organisms undergo anhydrobiosis, in which the near complete absence of water downshifts metabolism to a state of dormancy (117). Upon rehydration, metabolism and activity ensue. Like acute cell shrinkage, desiccation will induce crowding while increasing osmotic pressure and intracellular ionic strength (117). Thus, it is perhaps not surprising that intrinsically disordered proteins that are prone to crowding-induced regulation have been implicated in desiccation tolerance. In tardigrades, for example, IDR-containing cytoplasmic heat soluble proteins are necessary for survival and undergo gelation phase transitions (118). In contrast, *Arabidopsis* FLOE1, a disordered protein with prion-like features that are key for desiccation tolerance, is spread diffusely throughout the cytosol during the dry state, but it forms liquid-like condensates upon rehydration (119). This suggests that intrinsically disordered proteins are important for desiccation tolerance but can exhibit a diversity of yet-to-be elucidated phase behaviors that confer function. Desiccation represents an extreme physiological stress, but understanding how crowding-induced signaling facilitates adaptation under such conditions has important implications for controlling the physiology of biological systems subjected to the threat of climate change.

### Mechanical Compression

Physical force scan compress cells to the point where their internal contents become crowded. One example of this is cellular spreading, in which the flattening of adherent cells on a stiff support creates traction forces that trigger water efflux, increasing cell stiffness and cytoplasmic crowding (120). In this scenario, cell volume contraction is likely dependent on mechanosensitive channels that influence ion and water transport (118, 121, 122). Thus, the same RVI systems that activate during hypertonic stress may also participate in crowding-dependent cellular responses that arise from mechanical compression. Consistent with effects on complex cellular functions, physical compression-induced crowding caused by cell spreading was shown to influence stem cell differentiation (120).

Cells that are actively dividing and growing in confined spaces undergo compressive stress. Growth-induced pressure increases intracellular crowding, and this is associated with an inhibition of protein expression and cellular growth (123). This suggests a feedback mechanism that ties mechanical compression and molecular crowding to multicellular architecture. In proliferating cells growing unabated in confinement, severe compressive forces can be transduced to the nucleus, which can deform, resulting in nucllear envelope rupture, nucleocytoplasmic mixing, and DNA damage (30, 124, 125). These crowding-induced sequelae have implications for disease progression in malignancy and in proliferative disorders such as cystic kidney diseases.

### Cellular Senescence

Aging also influences intracellular molecular crowding. As cells persist over time, they swell and their cytoplasm undergoes dilution due to a lack biosynthetic scaling in association with cell volume (126). The lack of crowding agent biosynthesis diminishes the efficiency of cellular reactions and thus leads to senescence. Understanding the crowding-dependent mechanisms that mediate or prevent cellular senescence will be essential for future studies of long-term effects of molecular crowding on cellular physiology in the context of aging.

## CONCLUDING REMARKS

Despite long-standing interest in its biophysical properties, the relationship between cytoplasmic molecular crowding and physiology is just beginning to be appreciated. The role of crowding in functional processes largely remains obscure due to a lack of understanding of how cell density is sensed and controlled and how overcrowding can influence cellular functions. Fundamentally, these knowledge gaps stem from our inability to accurately measure crowding. Approaches that can assess the density and rheologic properties of the cytosol have been developed (28, 127–130), but some of these techniques may be challenging to adapt for studies in complex cell types, tissues, and whole animals, and others will have to be optimized (131). Indeed, in recent years, progress has been made in the development of in vivo sensors that can report crowdedness (132,133). Whether these tools can be adapted for use in animal models commonly used by the physiologist, however, remains an open question. The growing body of evidence demonstrating a close relationship between crowding and PS indicates that diverse biological processes are influenced by crowdedness. As our understanding of the functional significance of biomolecular condensates becomes clearer, the role of molecular crowding in physiology will also be unraveled.

## SUMMARY POINTS

1. The cytoplasm is crowded with macromolecules that can influence intracellular chemical reaction rates, water potential, and protein complex formation.
2. To defend against deleterious overcrowding, cells spend considerable energy to tightly regulate cell fluid volume, size, and macromolecule concentration.
3. Phase separation, involving the demixing of proteins and nucleic acids into membraneless biomolecular condensates, is a fundamental way that cells sense and respond to macromolecular crowding.
4. Diverse physiologic processes appear to be linked to crowding, including desiccation, transepithelial transport, aging, circadian rhythm, and central osmosensing, among others.

## FUTURE ISSUES

1. Biomolecular condensates likely play diverse roles in crowding-induced cellular responses. The underlying mechanisms that drive the formation and regulation of these structures and their downstream functional effects on physiology require further investigation.
2. Tools that permit dynamic measurements of crowding in response to environmental stressors are actively being developed and optimized for in vivo use. Such approaches will be necessary to draw clear connections between molecular crowding and physiology.
